# Ranked retrieval of Computational Biology models

**DOI:** 10.1186/1471-2105-11-423

**Published:** 2010-08-11

**Authors:** Ron Henkel, Lukas Endler, Andre Peters, Nicolas Le Novère, Dagmar Waltemath

**Affiliations:** 1 Database and Information Systems, University of Rostock, Rostock, Germany; 2 Computational Neurobiology, European Bioinformatics Institute, Hinxton, UK

## Abstract

**Background:**

The study of biological systems demands computational support. If targeting a biological problem, the reuse of existing computational models can save time and effort. Deciding for potentially suitable models, however, becomes more challenging with the increasing number of computational models available, and even more when considering the models' growing complexity. Firstly, among a set of potential model candidates it is difficult to decide for the model that best suits ones needs. Secondly, it is hard to grasp the nature of an unknown model listed in a search result set, and to judge how well it fits for the particular problem one has in mind.

**Results:**

Here we present an improved search approach for computational models of biological processes. It is based on existing retrieval and ranking methods from Information Retrieval. The approach incorporates annotations suggested by MIRIAM, and additional meta-information. It is now part of the search engine of BioModels Database, a standard repository for computational models.

**Conclusions:**

The introduced concept and implementation are, to our knowledge, the first application of Information Retrieval techniques on model search in Computational Systems Biology. Using the example of BioModels Database, it was shown that the approach is feasible and extends the current possibilities to search for relevant models. The advantages of our system over existing solutions are that we incorporate a rich set of meta-information, and that we provide the user with a relevance ranking of the models found for a query. Better search capabilities in model databases are expected to have a positive effect on the reuse of existing models.

## Background

### Importance of model exchange and reuse

**Figure 1:**
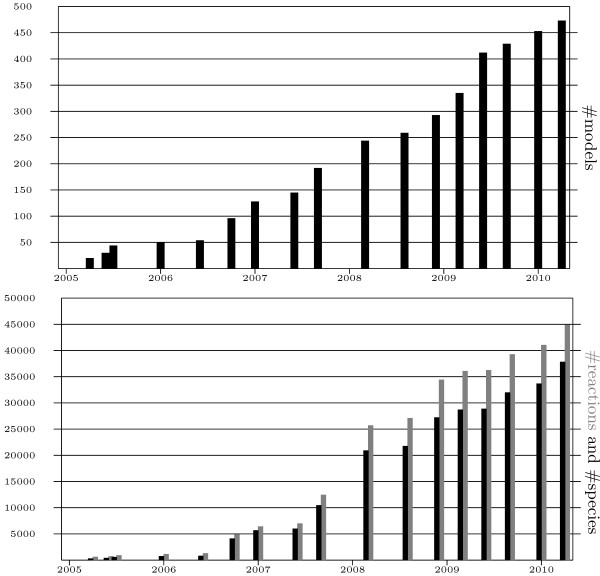
**Growing number of Computational Biology models and their components in BioModels Database**. Upper chart: Numbers of models in BioModels Database, as of January 2010. Lower chart: Number of species (black bar) and reactions (gray bar) in BioModels Database, as of January 2010. BioModels Database started with 20 models and a total of 322 species when it was launched in April 2005. In 2007 it already reached almost 200 models and 10482 species. The release in January 2010 recorded 453 models with 33702 species and 41069 reactions.

The study of a complex biological system now frequently includes the use of modelling and simulation techniques, in order to help understanding the system of interest, and to provide suggestions for promising experimental procedures [1]. The rising complexity of *modelled *systems (see Figure 1, number of encoded species and reactions in BioModels Database [2]), and the fact that research activities overlap between different research groups demand for model reuse. Modellers do not want, or cannot build their models of biological systems from scratch, but, on the contrary, need to seek for existing bits and pieces to build their models on, especially when composing complex systems by combining smaller sub-models (see for example [3,4]).

Standard formats for model exchange and open model repositories are crucial tools to make existing models available and accessible to the community as it becomes impossible to actually be aware of all existing models, and research groups involved in the modelling of a system of interest. Some standard formats developed for model representation are widely accepted. Examples include the *Systems Biology Markup Language *(SBML, [5]), CellML [6], or BioPAX [7]. Computational models of biological systems (bio-models) in standardised representation formats are available from different model repositories, including BioModels Database [2], the JWS Online Model Database [8], or the CellML Model Repository [9].

However, although getting more frequent, model reuse is not yet common-place. The reasons are similar to those hampering code reuse in computer science, where insufficient code documentation and missing modularisation have been the biggest hindrances [10]. Most models are created using computational modelling environments; the constituents' names are often generated automatically and therefore are semantically poor. Models with unspecific species names such as P*o*1, P*o*2, P*c*1, P*c*2 (for instance, see model BIOMD0000000060 in BioModels Database), or unspecific reaction names *re*1 to *re*76 (model BIOMD0000000227 in BioModels Database) are common-place. A documentation of the names' meaning, amongst other things, is essential.

### Standardised meta-information representation helps grasping models' nature

To countervail the problems experienced in computer science, efforts for the documentation of models' nature were developed. A minimum set of meta-information that is requested to be provided by many journals with each published bio-model is the *Minimum Information Required in the Annotation of a Model *(MIRIAM, [11]). Such meta-information provides a better understanding of a bio-model's complex and diverse *semantics *and, if computationally processed, enhances the model reuse.

MIRIAM meta-information encompasses general information about the model itself, e. g. the model's name, authors, or publication reference. But it also includes detailed descriptions of the model constituents, including the identification of encoded species, reactions, and compartments. MIRIAM itself is a textual recommendation, in form of a *Minimum Information *guideline following the MIBBI idea of coherent reporting guidelines for biological and biomedical investigations [12].

A technical, standardised way of providing the MIRIAM-recommended meta-information is the *MIRIAM standard annotation *[11,13]. The proposed format is a triplet referencing a piece of meta-information, also referred to as *annotation*, in an external resource. The reference to that meta-information is build of (1) the data type, (2) the identifier, and (3) a qualifier from a set of pre-defined qualifiers. Here the *data type *specifies the namespace within which to interpret the identifier. Some resources encode their knowledge as controlled vocabulary or ontologies. Among existing ontologies that are also used as data types by the MIRIAM standard are the *Systems Biology Ontology *(SBO, [14]), the *Gene Ontology *(GO, [15]), or the NCBI Taxonomy http://www.ncbi.nlm.nih.gov/Taxonomy/. One advantage of using ontologies, i. e. "explicit specifications of a conceptualization" [16], over free text information is the standardised encoding of biological knowledge that is then put into relation with other ontology terms. The MIRIAM standard *identifier *refers to the actual entry within the data type. It corresponds to the identifier (ID) the entry has in the external resource. Finally, the *qualifier *is used to characterise the relation between the annotated model element and the encoded meta-information. The possible qualifiers are defined at BioModels.net and include relationships such as is, isVersionOf, or hasPart[14].

For example, a species element encoded in a particular SBML model could stand for the compound "phosphosphoenolpyruvate" and in the model simply be called "PEP", offering little valuable information to the user. This compound, on the other hand, is described by the entry CHEBI:18021 in the *Chemical Entities of Biological Interest* (ChEBI, [17]) ontology. Referring to this particular identifier in that data resource by linking the resource and ID to the species element via the qualifier is, gives software and users access to a wealth of information independent of the elements name, such as synonyms, molecular and structural formulae and cross-links to other databases. Technically, the link is encoded in a standard form using URNs, e. g. urn:miriam:obo.chebi:CHEBI%3A18021 for the given annotation. Another example is the annotation of a reaction element in an SBML document. Given a reaction element in a particular model stands for the "phosphorylation of glucose by hexokinase during glycolysis". This enzymatic reaction is also described by the GeneOntology entry GO:0004396 (hexokinase activity). Attaching the URN urn:miriam:obo.go:GO%3A0004396 to the reaction element using the qualifier isVersionOf, semantically enriches it and again gives access to further information, like alternative terms and enzyme nomenclature codes.

#### Extending the MIRIAM information

In order to enable a fine-grained retrieval of bio-models, [18] proposes to consider even more information than MIRIAM's required one. Among them are versioning information on both the model and its annotations, but also information on the model encoding format, and information that is only related to the model, such as model behavior under certain conditions, simulation experiments applicable to the model, or simulation results available for the model. A detailed description of different kinds of meta-information considered in this work, even beyond MIRIAM is given in [19].

### Finding models in model repositories using Information Retrieval techniques

We argued that a crucial step for a computational system to return relevant models upon a user's query is the availability - and then incorporation - of meta-information on top of a model's structure [18]. With the advent and growth of Computational Systems Biology research, the number of bio-models available rapidly increases. For example, the number of bio-models available from BioModels Database is steadily growing, doubling about every 18 month (see Figure 1, number of models in BioModels Database). As a consequence, searching an existing model base for relevant models can result in a rather big number of models. Therefore, it is very important to support the user in *finding relevant *models in existing resources. It is common-place to leave the user with an unordered result set of models, without any explanation of why a particular model was found. For complex models the user is typically unable to grasp the model's nature at first sight [18]. Having no information to assess *how good *a model matched his query, he cannot decide on its relevance. *Information Retrieval *techniques, which have been widely and successfully used in other areas, offer exactly these benefits for bio-model retrieval.

Information Retrieval is "the process to recover an information stored in a system (i. e. a database) on users demand" [20]. One application for which the successful ranked retrieval of annotated documents has already been shown is *Multimedia Information Retrieval *(MIR). MIR models describe songs, images or videos annotated with different kinds of information, including meta-information like author or title, but also temporal or spectral information, as well as keywords. Currently, MIR distinguishes three independent classes of similarity measures depending on the kinds of identified features [21]:

**Metadata-based similarity measure (MBSM) **defines queries by connecting keywords gained from the media object with Boolean operators like ⋀, ⋁. Text retrieval techniques are then used to compare these query keywords with features of the multimedia objects.

**Content-based similarity measure (CBSM) **utilizes so-called low-level features, i e. automatically extractable items, such as rhythm. Queries make use of these features to search the content of music pieces. Different methods have been developed to retrieve the items represented by low-level features, e. g. humming, tapping or query-by-example.

**Semantic-description-based similarity measure (SDSM) **evaluates meta-information on multimedia objects that are described with predefined words of different vocabularies.

Motivated by the above observations, we propose a novel retrieval and ranking framework that takes into account different model meta-information to perform similarity-measure-based operations on bio-models. We are aware that *data *retrieval techniques have already successfully been applied to Life Science data in general [22]. Existing approaches do, however, not consider the retrieval and ranking of *models*.

## Results and discussion

Here we apply an adapted version of the aforementioned solutions for MIR on bio-model retrieval. To re-use MBSM for bio-model retrieval, the MIRIAM required meta-information on the model and its constituents is essential. Furthermore, we use parts of the meta-information suggested by [19] and [18]. When adapting CBSM techniques to bio-model retrieval, low level features (such as the encoded species, reactions, and so on) can be used. Finally, SDSM techniques can be used by tagging the models manually with relevant terms.

### Definitions

Our study necessitates a collection of *k *models from a pool of bio-models *M *and associated meta-information that is sufficient to rank the retrieved results with respect to a user's query. An annotated bio-model is defined as:

**Definition 1 **(Annotated bio-model). *An annotated bio-model m *∈ *M is described as a tuple m = (m_S_, m_A_) of*

1. model source code m_S _in a machine-readable format

*2. annotation information m_A _describing the nature of a bio-model, and of its constituents*.

In the following, we will not distinguish annotations of the model *m *from annotations of the model's constituents. All annotations will be processed equally, denoted as *m_A_*. The annotation information *m_A _*might be referred to as third party knowledge linked to *m_S_*.

A feature is defined as:

**Definition 2 **(Feature). *A feature f *∈ *F is an attribute or aspect of a model m instantiated either through its model encoding m_S _or its annotation information m_A_*.

**Definition 3 **(Term). *Let T be a set of words called terms, then P(T)={ρ:ρ⊆T} is the set of all subsets of T called power set*.

A model collection is then:

**Definition 4 **(Model collection). *A model collection C_M _is a representation of M. Each m_j _*∈ *M can be mapped on a c_j _*∈ *C_M _by splitting the model m_j _into features f *∈ *F and their instances $\rho_{f}\in P(T)$. So $c_{j}=\{(f_{1},\rho_{f_{1}}),\;.\;.\;.\;,(f_{n},\rho_{f_{n}})\}.$*

Those Definitions (1, 2, 3, 4) hold for each model *m_j _*∈ *M *classified into features and represented by *c_j _*∈ *C_M_*.

We furthermore define a query as (definition 5):

**Definition 5 **(Query). *A query $q=\{q_{f_{1}},\;.\;.\;.\;,\;q_{f_{n}}\}\in Q$ is a set of query parts $q_{f}\in F\times P(T)$ with q_f _= *(*f, ρ_f _*); *f *∈ *F and $\rho_{f}\in P(T)$. All query parts q_f _of a query q are pairwise disjoint*.

*q *∈ *Q *represents the user query. The parts *q_f _*of *q *can either be mapped on the full set of defined features *F*, or on a subset of *F*.

Assuming a collection *C_M _*of processed models *M *and extracted model features *f_i_, ..., f_n _*∈ *F*, we now define bio-model retrieval.

**Definition 6 **(Bio-model retrieval based on [23]). *An Information Retrieval model is a quadruple (C_M_, Q, FW, R*(*q, c*)*) where*

1. C_M _is a feature-classified representation of M

*2. Q is a set of queries q, where each part q*_f∈*F *_∈ *q can be mapped on a f *∈ *F*

3. FW is a framework for model representations, queries and their relationships

*4. R*(*q, c*) *is a set of ranking functions defining an order among c *∈ *C_M _with regard to q*.

The framework *FW *realises the retrieval functionality. Each ranking function *r*, when applied to a query *q*, returns a ranked list of model representations *c*. The order of retrieved results is determined by the ranking function itself, the underlying collection and by the particular query. From the ranked list of feature-based model representations *c_j_*, we deduce the ranking of the corresponding models *m_j _*represented by *c_j_*.

### Conceptual architecture of the framework

**Figure 2:**
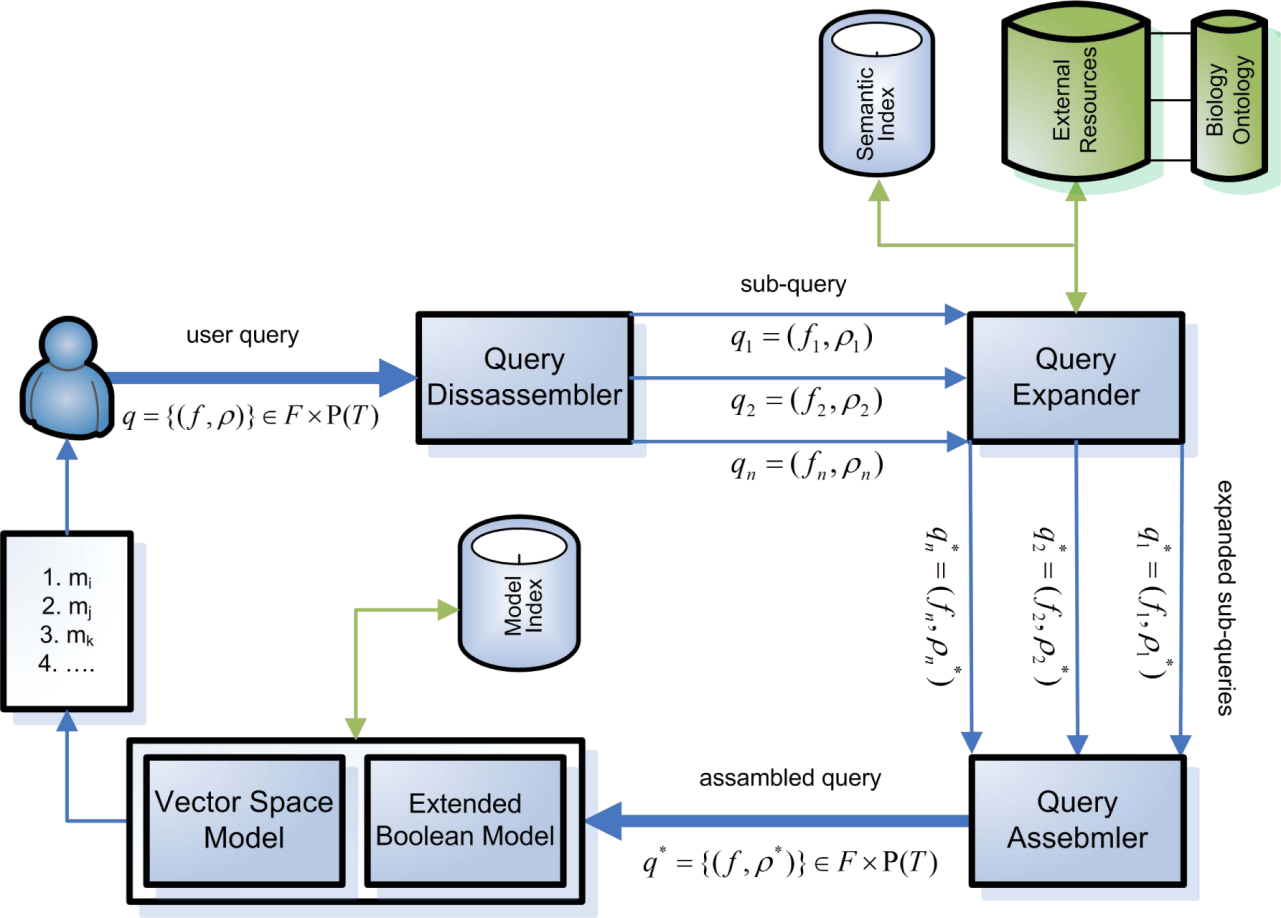
**Conceptual architecture**. Overview of the conceptual architecture of the proposed ranking- and retrieval system. A version has been implemented in BioModels Database. The architecture shows the process of transforming a *user given query *by creating sub-queries, which are then assembled by enrichment of structural information and semantic indexing (see also Figure 3). The re-assembled query is then sent to the retrieval and ranking module, which makes use of the Extended Boolean Model to retrieve a list of matching models, and the Vector Space Model to rank the list of retrieved models. To determine the ranking, different weight information is used. Those are, however, not shown in the given Figure.

To perform ranked retrieval of annotated bio-models, we use a combination of text retrieval, ontologies, simulation dependent data, and model meta-data. The conceptual architecture for the developed retrieval and ranking framework is shown in Figure 2.

For a user-given query *q*, consisting of a set of feature-assigned terms (*f*, *ρ*), we return a ranked list of models. The ordered list of models *m_j _... m_k _*is inferred from the order that is defined by the ranking function *r*(*c_j_, q*) > ... >*r*(*c_k_*, *q*), where *c_j _*is the most relevant model representation with regard to the query *q *(see definition 6). To achieve this order, each query *q *is first disassembled into a set of sub-queries *q_1 _*to *q_n_*. Each sub-query *q_i _*now contains a set of terms that will be mapped on a particular feature *f_i_*. However, the query parts *q_i _*are not directly executed on the data resources, but rather expanded using the Query Expander. So far we distinguish two different kinds of sub-queries:

**Semantic sub-query **is any query addressing model constituents. This type of query is applied to the SEMANTIC INDEX.

**Ontology sub-query **is any query enriching the user query by finding related ontological terms. This type of query is applied to the BIOLOGY ONTOLOGIES.

All expanded sub-queries are assembled into a final query q* which is sent to the retrieval and ranking system. The Extended Boolean Model [23] is used to select all models that are relevant to the query, and then the Vector Space Model [24] is used to define the ranking on those models. Both IR models work on the MODEL INDEX which contains all models and their associated URIs. The result of the process is a ranked list of model IDs.

### Architectural components of the framework

#### Types of user queries

We process and store information from different resources, and map them on our internal structures; i. e. full-text indexes and databases. As a result it becomes feasible to answer very specific queries. We distinguish two different types of queries. A query may consist of a number of terms (*query by value, QBV*) or of a complete set of features representing a model (*query by model example, QBME*).

**Query by value (QBV) **Using QBV, the user query *q *consists of features and free-text terms (*f*, *ρ*). The user given features *f *are a subset of all available features *F*.

**Query by model example (QBME) **Using QBME, a model forms the basis of a search for similar results, i. e. the complete set of features *F *is aligned.

Questions a user might have in mind are "Which models describe calcium concentrations in pancreatic cells?" (QBV), or "Are there any models dealing with the effects of caffeine on blood pressure in humans?" (QBV). One could also easily imagine to search for a model that "is similar to model BIOMD0000000227" (QBME).

#### Model index: incorporating model meta-information

The MODEL INDEX contains references to all models *m_i _*∈ *M*, as well as encoded information about constituents and meta-information.

Relevant features representing a bio-model were defined and grouped into several content-related *dimensions *to facilitate the creation of the bio-model collection *C_M_*. Each of those dimensions has a certain importance associated to it, i. e. a measure of how relevant the information it carries is (see Table 1). *(1) Model constituents *is an important dimension which contains several features describing a model's constituents, e. g. species or reactions. *(2) *Information about authors, encoders or submitters of a model are grouped into a *persons *dimension. *(3) *Publications or published abstracts are contained in the *publication *dimension. The *(4) user generated content *holds information like keywords or tags. To restrict search results timewise a *(5) dates *dimension holds time information, for example submission or modification dates. Finally, the *(6) administrative data *dimension contains specific information about the model file or the representation format used to encode the model.

**Table 1:** Importance of different information dimensions

dim	Part	Importance	Description
1	Administrative data	low	administrative data like id, file name, file version, encoding formalism
2	Persons	medium	covers the author, encoder and submitter
3	Dates	low	submission or modification date
4	Publication	high	main publication or description of the model
5	Constituents	very high	information about the model constituents
6	User generated content	very high	additional user-provided information, e. g. keywords

Information dimensions sorted by relevance. The information that is relevant for the characterisation of a bio- model's ranking is grouped into six different dimensions (dim). Each dimension has a different influence on the ranking. The least important dimension is the *administrative data*, the most important dimensions are the one encoding information about the model *constituents *and the one created from *user generated contents*.

The concept of dimension is a rather general one. Each dimension can, however, be refined into *features f*. A full list of features that make up the model index for all aforementioned dimensions can be found in Table 2. For example, the dimension *model constituents *is split into several features, among them *species, compartment, reaction*. Limiting a query to certain model features allows a user to be more specific. For example, it is possible to restrict a query caffeine to the feature *species *- and to disregard a "tribute to caffeine for the writing" in the *publication *feature. The values for each defined feature can be automatically extracted from a bio-model *m *if *m *complies with the given model definition 1. The additional assignment of weights for each distinct feature helps to determine similarity values, as will be explained later.

**Table 2:** Assigned feature weights by dimension

Dimension	Feature	Weight
Constituents		
(description)	modelName	4
	species	3
	compartment	3
	reaction	3
	parameter	1.5
	event	1.5
	function	1.5
	modelDescription	0.5

(URI)	modelURI	5
	speciesURI	5
	compartmentURI	5
	reactionURI	5
	parameterURI	3
	eventURI	3
	functionURI	3

Persons	Author	4
	Encoder	1
	submitter	1

Publications	publicationURI	5
	publicationText	2.5

	content	1
User generated		
Content	-	-

Dates	CreationDate	1
	modificationDate	1

Administrative data	ID	1
	additionalID	1
	path	1
	content	1

Feature weights for the different model dimensions. Each dimension is further separated into the features it covers. For each feature, a concrete relevance value, i. e. weight, is given. For example, in the *Constituents *dimension, one important feature for the model description is the modelName. The different URIs (modelURI, speciesURI, compartmentURI and reactionURI) also play an important role in determining the ranking. A less influential feature is the modelDescription, as for example found in the SBML <notes> tag.

#### Semantic index: identifying biological entities

Bio-model entities can be described by annotation information *m_A _*encoded in MIRIAM standard URIs and stored in the Model Index. When searching for a model, a user cannot be expected to know the URIs for each biological entity of interest. On the contrary, searches for a constituent or bio-model must be possible using *characterising terms*, i. e. keywords. Therefore, the URIs must be parsed and the extracted information processed. The textual representation of each known constituent found in the external resources is resolved from its URI, and then indexed. By making it available for searching, *keywords *describing a model constituent can be used to retrieve models. For example, when searching for models dealing with caffeine, one may type either caffeine, 1,3,7-trimethylpurine-2,6-dione, or even C_8_H_10_N_4_O_2_.

To map the textual descriptions, and also synonyms of a term, on a set of URIs representing the best matches for a defining term, a so-called SEMANTIC INDEX is used (see Table 3 for the structure of the Semantic Index). This index contains all URIs found in the models included. It furthermore is build of a column for each existing qualifier. Every model *m *that contains a particular URI is added to the set of model IDs in the relevant qualifier column. The semantic index therefore enables to link a URI, resolved from search terms, to a set of bio-models within the collection *C_M_*.

**Table 3:** Semantic index

URI		qualifier		content
		
	bqbiol_is	bqbiol_isVersionOf	bqmodel_is	
urn:miriam:obo.chebi. CHEBI:27732	BIOMD0000000241	BIOMD0000000241		caffeine chebi 27732 chebi home advanced search browse ontology periodic ... moleculeschebimain caffeine chebi 116485 central nervous system stimulant caffeine ryanodine receptor modulator mutagen 1,3,7-trimethyl-3,7 dihydro-1 h-purine-2,6 iuphar 1,3,7-trimethylxanthine dion msdchem d00528 kegg drug [..]

urn:miriam:kegg. compound:C07481	BIOMD0000000241		BIOMD0000000241	kegg compound c07481 entry c07481 compound name caffeine 1,3,7-trimethylxanthine formula c8h10n4o2 mass 194.0804 structure remark d00528 comment source coffea arabica tax 13443 xanthines reaction r07920 r07921 27732 knapsack c00001492 [..]

urn:miriam:kegg. compound:C00385	BIOMD0000000015			kegg compound c00385 name xanthine formula c5h4n4o2 mass 152.0334 ko00230 purine metabolism caffeine metabolism [...]

urn:miriam:kegg. compound:C00048		BIOMD0000000221 BIOMD0000000222 BIOMD0000000219 BIOMD0000000218		kegg compound c00048 entry c00048 glyoxylate glyoxylic acid formula c2h2o3 mass 74.0004 structure reaction r00013 r00364 purine metabolism path ko00232 caffeine metabolism glycine serine null_1 threonine metabolism [...]

. . .				. . .

The semantic index is used to connect each existing URI in the database to the models in which it occurs. Thus, each column contains a set of IDs identifying a bio-model in *C_M_*. We additionally store *how *the URI is connected to the annotated model constituent (through the *qualifier *column). For each URI, the content, i. e. textual representation, that had been extracted from the ontology term corresponding to the URI, is normalised and indexed as well. A query can then be enriched by further related URIs (see also Figure 2, Ontology Query), resulting in an expanded query.

Having build the Semantic Index, queries may now be limited to models that use a particular qualifier to link a constituent to an annotation. For example, a user searching for caffeine can limit the result to models qualifying the annotation with is and isHomolog. The models using the query term in conjunction with is could be ranked higher. This procedure also allows for weighting URIs differently according to their associated qualifiers.

The result of a query on the Semantic Index is a weighted, ranked list of URIs for each query term. That list is passed on to the Model Index where it represents a sub-query result that together with other sub-query results is assembled into a similarity value.

#### Biology ontology: incorporating similar constituents

Sometimes it might be useful to also include models with constituents that are *similar*, though not identical, to the one described by the original search terms, for example, if a search resulted in only a few models containing a particular constituent. BIOLOGY ONTOLOGIES expand a query by deriving similar constituents. A user searching for models encoding the constituent caffeine may also be interested in models containing the constituent xanthine, which is structurally related to caffeine.

To compare the relevance of a search term with terms in a particular ontology, we use a solution proposed by Schulz, Liebermeister (discussed in personal communication), who suggest to map different ontology Web resources on one common ontology. Using that ontology, the similarities of ontology terms are measured. The approach also takes into account different relations between the terms. In our work, we used that approach to compute weights for ontology entries within a certain range of a given term. Apart from that method, other works from IR research exist which might be incorporated in later studies, e. g. [25].

#### Incorporating weights

After retrieval, the relevant bio-models are ranked. The ranking function comprises weights derived from different sources. (1) The MODEL INDEX itself is used to incorporate weights derived from IR techniques such as term frequency - inverse document frequency [23]. (2) The importance of each feature is expressed by its weight (see Table 2). (3) A user may in addition assign a weight to a term in the query in order to increase that term's importance. (4) Preliminary results of the single sub-queries assigned to particular data resources are evaluated. (5) Weights derived from ontologies (see BIOLOGY ONTOLOGIES) may change the result ranking, e. g. models retrieved by ontologically derived terms can be ranked lower than others.

#### Ranking the results

All weights assigned to a model are used to determine the model's position in the vector space that is spanned by the Vector Space Model. Having all model positions identified the similarity can then be computed and the ranking inferred, based on the models' positions.

### Implementation: enabling model retrieval in BioModels Database

The introduced implementation is based on prior work on a general framework for testing different ranking functions on a given model base, called Sombi http://sourceforge.net/projects/sombi.

Here we present an implementation for BioModels Database. We assume that the model source code *m_S _*is provided in the open, standardised model representation format SBML. Furthermore, annotations *m_A _*should be encoded using the MIRIAM standard annotation, i. e. MIRIAM URIs. The implementation is based on the architecture presented in the previous section. All source code is freely available from the Biomodels.net SVN Sourceforge repository https://biomodels.svn.sourceforge.net. The retrieval and ranking system is available online at http://www.ebi.ac.uk/biomodels-demo/.

The advantage of using BioModels Database as a proof of concept lies in the amount of stored models - currently 241 curated, i. e. verified, models and additional 213 non-curated models (as of 2010-04-01). All models are encoded in SBML. All models in the curated branch are annotated, and as a consequence provide sufficient meta-information for a thorough testing of the ranking and retrieval system.

Furthermore, analysing the stored information together with the BioModels.net team led to tentative weights for the different features (see Table 2), and helped on pinpointing the importance of different qualifiers (shown in Table 4).

**Table 4:** Qualifiers and their assigned importance

Qualifier	Weight
is	2.0
isHomologTo	1.7
hasPart	1.5
isPartOf	1.5
isVersionOf	1.5
hasVersion	1.5
isEncodedBy	1.3
isDerivedFrom	1.3
encodes	1.3
isDescribedBy	1.0
occursIn	1.0
hasProperty	1.0
isPropertyOf	1.0

The table shows the different qualifiers available from MIRIAM resources. The qualifiers are used in the SEMANTIC INDEX. Each qualifier has a particular weight assigned to it which reflects the strength of connection between a URI and a constituent.

We extend the current BioModels Database search engine by including a greater number of features in the search process, by weighting different information, and by ranking the results according to the user query. Both types of queries, QBV and QBME are supported. The model index contains 454 models with 140977 terms separated into 25 features. The SEMANTIC INDEX contains 2261 URIs with 409124 terms. The used BIOLOGY ONTOLOGIES are NCBI Taxonomy, GO, ChEBI, KEGG Compound and KEGG Reaction [26] (as of 2010-04-14). We anticipate to include more formal (biological) semantics in future versions, and to turn them into additional features for the similarity measure. Candidates for information relevant to preserve a bio-model's semantics have been suggested in [19].

The *Lucene Framework *[27] is integrated in the search system to create, maintain and search both the Model and Semantic Index. It provides retrieval functionality based on the Extended Boolean Model; its ranking possibilities are based on the Vector Space Model. To implement the retrieval and ranking process described above, Lucene has been extended by the different indices and sources, e. g. the Semantic Index. While the implementation makes use of an adapted Lucene built-in similarity function, it will be useful in the future to provide advanced users of the ranking system with a collection of different similarity functions to choose from.

#### Search engine possibilities

**Query by value **Query by value allows the user to either perform a free text search querying all features, or a more sophisticated search selecting features of the different dimensions to be searched (refer to Tables 1 and 2). For instance a user is able to search for models having a certain author or for models including a particular "species". Furthermore, it allows to weight the different parts of a user's query using the specific *feature matrix *shown in Table 2.

Depending on the dimension selected, the query might be enriched or limited. This is especially important for the constituent dimension. For example, different terms describing a model constituent are used to query the SEMANTIC INDEX. The result is a list of weighted URIs, which is then used to identify a model in *C_M _*in case the model itself does not provide the search terms the user queried. When searching a model by URI, the importance of an URI within the model is reflected through a qualifier; i. e. models encoding a URI with the qualifier is are more important than models encoding the same URI with the qualifier isVersionOf. The weighting is done using the qualifier matrix shown in Table 4.

Additionally, the user is able to vary the importance of his search terms; i. e. one term describing a constituent can be more important than another. This *weight *is taken into account when computing the ranking. Besides the sophisticated ranking and retrieval system, the search engine supports common IR techniques like fuzzy search, range or proximity search, as well as wild-cards or phrase search [23].

**Query by model example **When querying by model example, the model used as a bait is analysed, and the values of extracted features are queried against the bio-model collection *C_M_*. A ranked list of best matching models is retrieved. Enriched queries are switched off, as the example model itself provides sufficient contextual information.

### An example for model retrieval and ranking

The following example illustrates the functioning of the reference implementation. We want to search for *recent models by non-bogus authors describing the effect of caffeine in human's digestive tract when drinking a cup of coffee*. The characteristics fulfilled by the resulting models are:

1. the model *should have *the compartment gut encoded

2. at least one species *must be *exactly caffeine (qualified using is)

3. the model *should *have been submitted later than 2008

4. the author of the reference publication *must not *be John Doe

**Figure 3:**
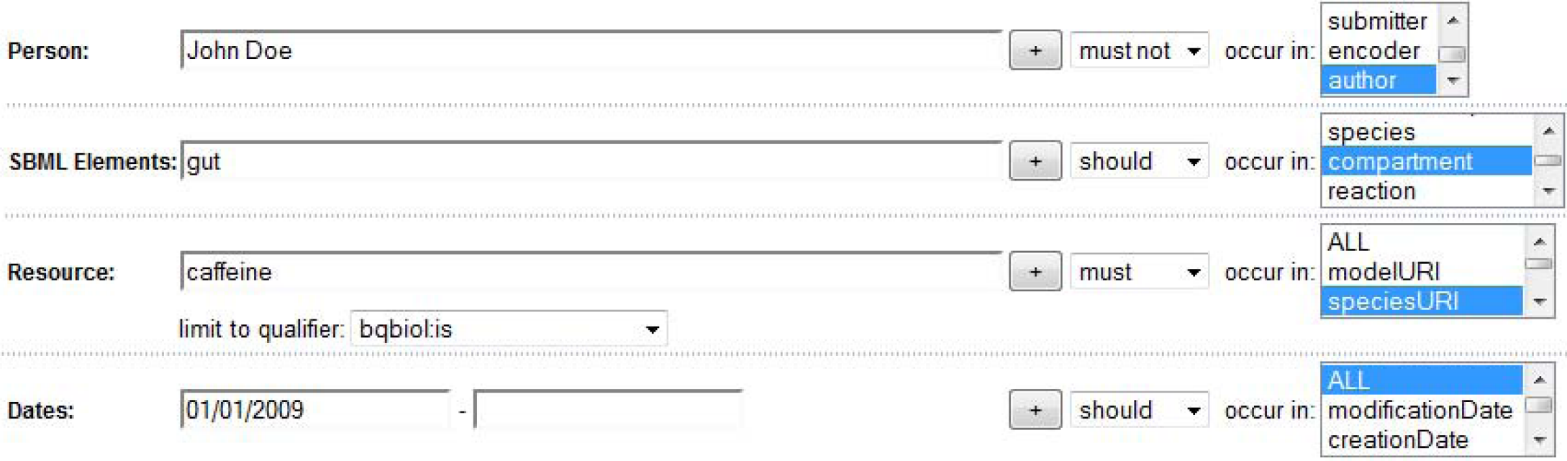
**Sample query on the new BioModels Database search interface**. Screenshot of a part of the new search interface of BioModels Database. The interface allows to search for *Persons, SBML elements, Resources*, and allows to restrict the search terms to particular features using a single qualifier. Models may only be considered for a certain range of *dates*. The sample search correspond to *recent models by non-bogus authors describing the effect of caffeine in human's digestive tract when drinking coffee*.

That query can be submitted easily through the proposed advanced search interface of BioModels Database. The query is shown in Figure 3. The specification of different levels of requirements (should, must, must not) helps to be more specific in restricting the search.

To answer the query, the system first resolves the constituent caffeine into a set of URIs (SEMANTIC INDEX). Since the search for caffeine is restricted to the qualifier is (*must be exactly *caffeine), only the retrieved URIs that are linked to a model using the is qualifier are kept. Of those, a weighted list of URIs is build and then used for the feature speciesURI to query the MODEL INDEX. For our example, the three best matching URIs are (a) urn:miriam:obo.chebi:CHEBI%3A27732, (b) urn:miriam:kegg.compound:C07481 and (c) urn:miriam:kegg.compound:C00385. The URIs (a) and (b) both define caffeine, one in ChEBI [17] and one in KEGG [28]. The URI (c) describes xanthine, a chemical structurally related to caffeine.

Together with the queries for gut in the component feature and *not *John Doe in the author feature, the MODEL INDEX query is internally assembled to:

+speciesURI:( urn:miriam:obo.chebi:chebi%3A27732 ^0.82

              urn:miriam:kegg.compound:C07481 ^0.67

              urn:miriam:kegg.compound:C00385 ^0.55)

compartment:(gut)

-author:(John Doe)

date:([01/01/2009 - *])

The prefix + and - denotes if a feature *must *or *must not *occur, no prefix implies the feature *should *occur. The ˆ denotes the weight assigned to the sub-query results retrieved from the semantic index. We use the Extended Boolean Model to query the index for each feature independently (speciesURI, compartment, date and author). The preliminary results are four sets of matching internal model identifiers. These sets are then conjuncted using Boolean algebra and taking into account whether a feature *should*, *must *or *must not *occur.

**Figure 4:**
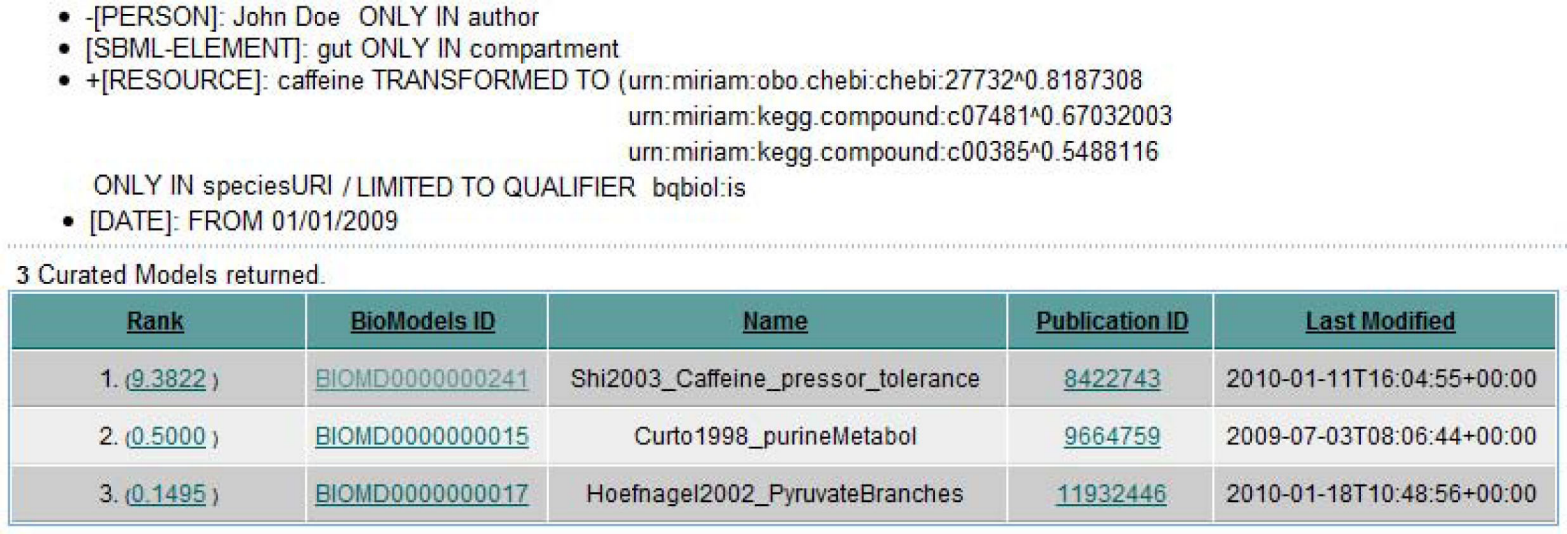
**Ranked results**. Search result obtained on BioModels Database with the given sample query (see Figure 3). The upper panel shows the enriched query. Due to the precise formulation of the query, and the requirement that caffeine must occur and additionally must be qualified with is, the result contains only three hits. (1) This model matches the top two constituents resolved by the semantic index, and additionally the term *gut *in the compartment feature. (2) The model matches the constituent ranked third by the semantic index. (3) The lowest ranked model only matches one constituent ranked eight by the semantic index - this is a very weak relation resulting in a very low rank.

In a second step, the results are ranked using the Vector Space Model, according to the different types of weights. The predefined feature weights (Table 2) put a particular importance on the speciesURI feature. Thus, all models that matched the speciesURI feature are ranked high, incorporating the weight created by the sub-query to the semantic index. If a retrieved model, besides the mandatory features (*must*), matches additional optional features (*should*), the scores are summed up, resulting in a higher rank. In this case, the feature "date" is not very important - thus, it results only in a small increase of a model's score if the feature matched. The ranked results for the sample query performed on BioModels Database is shown on Figure 4.

## Conclusions

This paper presents, to our knowledge for the first time, the application of Information Retrieval techniques on Computational Biology models. The theoretical method relies on knowledge extracted from model annotations, but also incorporates context information. The BioModels Database implementation presents a practical example of this method. It enhances significantly the search possibilities of BioModels Database users. Thorough evaluation, for instance using F-measures, is needed, but currently difficult due to the lack of reference to compare with. The concepts' generality ensures it is easy to apply to other models bases.

## Authors' contributions

The application of ranking and retrieval methods on bio-models based on model annotations was suggested by DW. AP, RH and DW discussed different similarity functions and set up the architecture for the Sombi system. RH implemented the approach in BioModels Database during his research stay at the EBI, supervised by NLN. LE and RH discussed and determined the different weights for features and qualifiers used for the similarity function. LE provided detailed examples for the evaluation of the approach. All authors contributed to the manuscript and all authors have read and approved the final manuscript.
